# Alternative delivery of male accessory gland products

**DOI:** 10.1186/1742-9994-11-32

**Published:** 2014-04-07

**Authors:** Z Valentina Zizzari, Irene Smolders, Joris M Koene

**Affiliations:** 1 Animal Ecology, Department of Ecological Science, VU University Amsterdam, De Boelelaan, 1085, Amsterdam 1081 HV, Netherlands; 2 Science Faculty, Radboud University Nijmegen, P.O. Box 9010, 6500 GL Nijmegen, Netherlands

**Keywords:** Injection, Mating strategy, Sperm transfer, Spermatophore, Sexual selection

## Abstract

To increase fertilization success, males transfer accessory gland products (Acps). Several species have evolved unconventional Acps transfer modes, meaning that Acps are transferred separately from the sperm. By surveying the sperm-free Acps transfer cases, we show that these animals have evolved a common strategy to deliver Acps: they all inject Acps directly through the partner’s body wall into the hemolymph. Our review of this mode of Acps transfer reveals another striking similarity: they all transfer sperm in packages or via the skin, which may leave little room for Acps transfer via the conventional route in seminal fluid. We synthesise the knowledge about the function, and the effects in the recipients, of the Acps found in the widely diverse taxa (including earthworms, sea slugs, terrestrial snails, scorpions and salamanders) that inject these substances. Despite the clearly independent evolution of the injection devices, these animals have evolved a common alternative strategy to get their partners to accept and/or use their sperm. Most importantly, the evolution of the injection devices for the delivery of Acps highlights how the latter are pivotal for male reproductive success and, hence, strongly influence sexual selection.

## Introduction

### The reproductive significance of male accessory gland proteins

Males employ legions of different pre-copulatory tactics [1] to increase their chances of fertilizing females’ eggs (e.g., ornaments, mate guarding, physical male-male competition, rival ejaculate removal, attraction via pheromones). However, finding a mate and achieving copulation does not suffice to ensure successful reproduction. In addition to pre-copulatory processes involved in finding and copulating with a mate, post-copulatory sexual selection can take place in the form of competition among sperm for the fertilization of eggs and/or cryptic female choice [2].

In internally fertilizing animals, post-copulatory sexual selection favours the evolution of male accessory gland agents that directly influence female physiology (see e.g., [3-7]). In copulating species such substances, together with the sperm and other non-sperm components, form the semen. Male accessory gland substances are produced by specialized glands and are generally referred to as seminal fluid proteins (especially when transferred together with the sperm; e.g., [7,8]) or accessory gland products (e.g., [9]). These substances are represented by a great array of peptides, proteins and other types of molecules that exert wide-ranging effects on female reproductive activity [4]. Hereafter, we will use the common abbreviation Acps (accessory gland products) to refer to this wide range of biological products secreted by male accessory glands.

Regardless of the bioactive molecule produced by the accessory glands, males of many species have been reported to gain benefits by transferring Acps during mating. Evidence for their presence and effects is abundant (e.g., [10]). For example, they can render a female unwilling or unable to remate for some time, thus facilitating sperm storage [11]. Acps can also modulate ovulation and/or oviposition, ensuring that any eggs laid will be fertilized by that male’s sperm [4,12]. By far the best-studied system, in which Acps provide clear reproductive fitness advantages to males, is the fruit fly *Drosophila melanogaster*. In this species numerous proteins have been shown to be transferred along with the sperm [13,14]. For example, the so-called sex peptide triggers increased egg laying and reduced sexual receptivity of females [8,15].

Sexual selection will most strongly drive the evolution of Acps when females (or hermaphroditic sperm recipients) mate promiscuously, store sperm and have specialized sperm-digesting organs. Thus, males (or hermaphroditic sperm donors) that manage to alter these processes to increase their sperm’s chances of being used will have a clear evolutionary advantage (reviewed in [16]). Many studies have shown that the transfer of Acps is widely distributed in the animal kingdom, from invertebrates [17,18] to vertebrates [19,20]. Substances that affect female reproduction are thus a widespread phenomenon, highlighting the importance of male accessory glands for successful male reproduction.

#### Diversity of Acps- and sperm-transfer modes

Animals with sexual reproduction exhibit two broad modes of fertilization. (1) In external fertilization, both male and female gametes are spawned into the environment (usually water). (2) In internal fertilization the sperm are transferred to the partner or the partner takes them up actively from the environment and fertilization occurs inside the egg-donor’s body.

Animals with internal fertilization exhibit two ways in which secretions from the accessory glands are transferred to the recipient: 1) Acps are carried in seminal fluid together with the sperm; 2) Acps are transferred separately from the sperm. Moreover, gametes are transferred to the recipient via several routes. Most notably, in many mating systems in the animal kingdom, insemination does not occur in the conventional way of using a copulatory organ to transfer the ejaculate into the female reproductive tract. Although sexual selection research has largely focused on male traits and sperm competition, surprisingly little focus is placed on the modes of sperm transfer, even though this could have great impact on the action of Acps. The modes of sperm transfer in animals with internal fertilization are broadly categorized as follows.

a. Spermatozoa are carried in unencased ejaculates, which contain sperm plus non-sperm components. Two types of transfer can be distinguished:

a1. Ejaculates are transferred via the highest level of bodily contact. The sperm donor uses a copulatory organ to deliver the sperm either into the sperm recipient’s reproductive tract (copulation *sensu stricto*) or into the hemolymph (i.e., hypodermic insemination).

a2. Ejaculates are transported via a skin groove. The sperm are transported over the outside of the body to the sperm recipient’s reproductive tract via a specialized groove and the sexes are involved in a more or less intimate contact.

b. Spermatozoa are encased in packages (spermatophores). Again, two types are possible:

b1. Spermatophores that contain sperm plus non-sperm components that act on female physiology and/or contribute to egg production [21,22]. The degree of contact is highly variable between species and shows a continuum from intimate bodily contact (e.g., lepidopterans [23] and orthopterans [24]) to no contact at all (e.g., [25-27]).

b2. Spermatophores that contain sperm plus non-sperm components without known effects on female physiology. The degree of contact is highly variable, but the mating behaviour generally involves contact between the two partners (see the review by [28] for a description of sperm-transfer modes in arthropods).

In categories a1 and b1 spermatozoa are accompanied by Acps acting on female physiology. In categories a2 and b2 such Acps have not been reported, although we cannot entirely rule out that non-sperm components are transferred along with the sperm.

Importantly, in order to induce a physiological change in the sperm receiver, such substances would need to exert their function prior to fertilization. However, the transport over the body along with the sperm and the encasing in the spermatophore would delay their activation or immediate effect on the receiver’s reproductive system, and make a paternity assurance strategy less efficient, especially in polyandrous species. Therefore, it seems unlikely that ejaculates transported via a skin or spermatophores would transfer Acps acting on female physiology to the receiver’s reproductive system. In addition, in the specific case of ejaculates transported externally, the exposure to the environment may hamper the efficiency of Acps as bioactive molecules’ stability strongly depends on pH (e.g., [29]). Does this mean that there is no opportunity for such males to increase their fertilization success using Acps? There are strong indications to the contrary.

In this review we focus on unconventional Acps transfer modes, that is Acps transferred in other ways than via the ejaculate. Males of species exhibiting this mode of Acps transfer have been reported to engage in an intimate contact with the partner before transferring their ejaculates via the skin or encased in spermatophores. Interestingly, our literature survey reveals that these species employ specialized structures in order to deliver the Acps and convince their partners to accept and/or use their sperm [16].

After building a list of taxa considered accredited cases of this type of Acps transfer, we assess the male substances transferred during courtship, and analyse those in the context of their fitness consequences for males as well as their recipients. We identify a set of morphological structures and derive their crucial function for the delivery of the above substances. Finally, we interpret the observations in light of new considerations about the importance of Acps for sexual selection. Our category of Acps injection resembles that given by [30] of *traumatic secretion transfer*. However, the category reported by [30], includes also the intragenital transfer (e.g., [31]), while our survey only includes species that inject Acps directly through the partner’s skin into the hemolymph.

It should be noted that dissociated sperm transfer (category b1 above) of soil arthropods [26,32] and sessile marine invertebrates [33], is not included in our survey. Our survey also excludes taxa displaying hypodermic insemination (category a1 above; e.g., leeches and bedbugs), in which the mating involves wounding the female tissue in order for the male to inject its ejaculate (reviewed in [30]). Though it has been hypothesized that antimicrobial ejaculate products may have a secondary gift function [34], whether males of these species generally inject only spermatozoa or also Acps has not been properly documented and goes beyond the scope of our categorization.

### Review

#### Alternative strategy to deliver male accessory gland proteins

**Figure 1:**
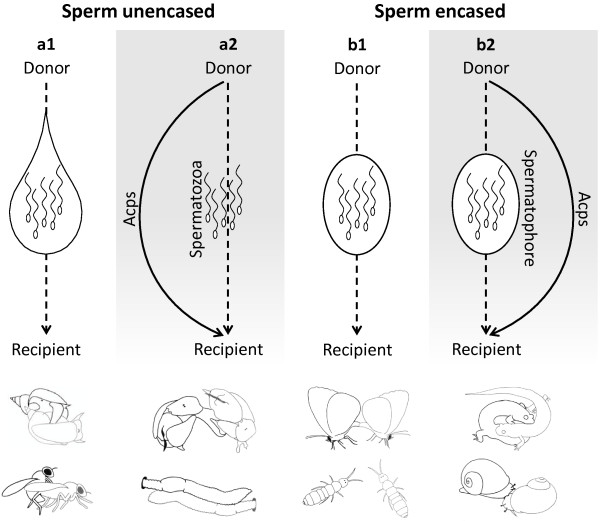
**Classification scheme to identify sperm-transfer modes for internally fertilizating species.** The labels a1, a2, b1 and b2 refer to the labels used in the text. The dashed arrows represent the introduction of sperm into the female system; the solid arrows represent the injection of Acps via the body wall. The sperm within the drop-shape represent semen (i.e., sperm plus non-sperm components); the sperm in the oval shape represent sperm encased in a spermatophore. Underneath each category, we have indicated several animal species that are known to use this mode of sperm and Acps transfer: a1, pond snails and fruit flies; a2, sea slugs and earthworms; b1, butterflies and springtails; b2, salamanders and land snails.

Animals with internal fertilization exhibit two ways in which Acps are transferred to the recipient. Acps can be carried in seminal fluid together with the sperm or transferred separate from the sperm. Crucially, conventionally copulating species that transfer an unencased ejaculate do not transfer their Acps in other ways than via the ejaculate. The occurrence of transfer of Acps separate from sperm is thus dependent on whether the ejaculate is transported externally over a groove on the body wall, transferred internally by means of spermatophores or deposited on the substrate (see Figure 1). By synthesising all the examples of Acps transfer separate from sperm we show that these animals have evolved a common strategy to deliver Acps: they all inject Acps directly through the partner’s body wall into the hemolymph. We synthesize our list of surveyed taxa according to the mode via which the spermatozoa are actually transferred.

#### Sperm transported externally over a groove on the body wall

**Earthworms** (Oligochaeta) are simultaneous hermaphrodites that deposit their sperm via external transport in or near the female gonopore [35-38] (category a2). In the earthworms *Eisenia fetida* and *Lumbricus terrestris* the spermatozoa are transported through a shallow groove-like depression (the seminal groove) in the sperm donor’s integument from the donor’s male genital openings to the receiver’s female spermathecae [36,37]. The depression of the groove moves rhythmically to transport the sperm to the clitellum, where the spermatozoa are transferred into the spermathecae of the recipient, where they are stored for later fertilization of a cocoon [36,37].

In *L. terrestris,* the sperm donors pierce their partners’ body wall numerous times with chitinous hairs called copulatory setae. Each of these special setae is equipped with a gland at its base that releases its products along the grooves of the seta. This piercing behaviour results in the injection of Acps into the sperm recipient [39]. One of the bioactive substances involved is ubiquitin, a small regulatory protein known for its role in tagging larger proteins for proteolysis [40]. The setal gland secretion has been demonstrated to increase the recipient’s sperm uptake and reduce its receptivity after copulation [38]. Both changes suggest that the sperm donor’s probability of fertilizing the eggs of the sperm recipient is increased [38].

Similar to earthworms, some **sea slugs** (Opisthobranchia) are known to transport their spermatozoa along the outside of their own body before transferring those to their partner [41,42] (category a2). In this case, the spermatozoa are transferred by means of the so-called penial papilla. When they reach the penial papilla, the papilla picks up the spermatozoa and guides them into the gonopore of the partner [43]. The cephalaspidean nudibranch *Siphopteron quadrispinosum*, a simultaneously hermaphroditic species, can mate via reciprocal or unilateral sperm transfer [44] and has a syringe-like penile stylet. This stylet is used to hypodermically inject prostate gland fluid into the body cavity of the partner [41,44]. During the precopulatory phase of mating both partners try to stab each other with their stylet, while avoiding their partner’s stylet. If one individual succeeds in stabbing first, it gets to mate unilaterally as a male, thus probably increasing its male reproductive success without having to invest in female reproduction [41]. It has been suggested that the injected Acps are responsible for the stabbed individual allowing the stabber to inseminate [41], but see [44].

#### Spermatophore transferred to the female

**Land snails** (Pulmonata), which are simultaneous hermaphrodites, exchange spermatophores by each transferring one into the receiver’s female tract (category b2). Prior to transfer, land snails perform a peculiar courtship behaviour during which the donor transfers Acps to the recipient: they stab a calcareous “love-dart” through the mating partner’s body wall (e.g., [45,46]). The love-dart is produced and stored in a sac called stylophore or dart sac [47]. During courtship, the muscular stylophore is forcefully everted and the calcareous dart is stabbed into the partner. After reciprocal dart shooting, the partners insert their penises and exchange spermatophores. For the brown garden snail *Cornu aspersum* (formerly *Helix aspersa*) it has been shown that the dart is covered by mucus, produced by the accessory glands that accompany the dart sac, which is transferred into the partner’s hemolymph [48]. Previous work found that snails hit by a love-dart stored significantly more sperm than snails that were not hit [49], which was subsequently shown to increase the donor’s paternity [50,51]. Crucially, this effect is not caused by the mechanical stimulation of the dart shooting but is mediated by the Acps present in the dart’s mucus [45].

Although the above findings are all related to *C. aspersum*, the mating and dart-shooting behaviour of Japanese land snails of the genus *Euhadra*[52] and the Cuban land snail *Polymita muscarum*[53] have also been studied. In contrast to *C. aspersum*, which shoots only one love-dart and leaves it in its partner’s body, *E. subnimbosa* and *P. muscarum* stab their partners repeatedly with the same love dart, which is smaller and simpler than *C. aspersum*’s and remains attached to the shooter’s dart sac [47,52,53]. Recent work on *E. quaesita* demonstrated that in this species the repeated stabbing results in delayed remating of the partner [54]. Finally, another example of a species that uses its dart repeatedly as an injection device was found in the genus *Everettia*[55]. These repeatedly-shooting species all transfer sperm afterwards in a spermatophore [52,53].

#### Spermatophore deposited on the substrate

In **scorpions** (Arachnida) sperm transfer takes place via a spermatophore that is attached to a solid surface by the male (category b2). Scorpion mating rituals are well documented for several species (e.g., *Hadrurus arizonensis*: [56]; *Bothriurus buecherli*: [57]; *Scorpiops luridus*: [58]). In the above species the male courtship behaviour consists of three main steps. (1) When male and female meet, they both rock and judder, though this is not restricted to reproductive behaviour. The actual mating behaviour starts when the male grabs the female’s pedipalps and the side of her abdomen. The male probes the areas with soft cuticle on the female with his sting and in one mating interaction the male can sting the female up to 14 times. This behaviour is referred to as the “sexual sting” [56,57]. (2) Subsequently, the male begins the next phase, the 'promenade a deux' (first described by [59]). He holds the female’s pedipalps and walks her around in several directions. This promenade continues until the male finds a suitable solid substrate for his spermatophore. (3) Once the spermatophore is deposited, the male pulls the female in such a position that the valves of the spermatophore touch her gonopore. Afterwards the male releases the female’s pedipalps and she walks away.

The substance that is delivered by the scorpion’s sting (which it also uses for defence against predators and to capture prey) is produced by a pair of venomous glands in the last postabdominal (tail) segment. Proteomic analyses of nine scorpion species from the family Buthidae have shown that the venom is a complex mixture with an average of a hundred different components, which differ among species [60]. Research on the scorpion’s venom has mostly focused on the neurotoxic components, with more than 350 different molecules described [60]. Components with other functions are Hadrurin, an antimicrobial and cytolytic peptide [61] and Hadrucalcine, a peptide capable of activating skeletal Ryanodine receptors [62]. Although the sexual sting has been suggested to subdue aggressive female behaviour [58], further research is clearly necessary to unravel the reason for the scorpion’s sexual sting, its effects on the female, and the identity of the active component(s) involved.

**Salamanders** and **newts** (Amphibia) are the only group of vertebrates in which the external transfer of spermatophores is known (category b2). During the sexual encounter, males of these species display typical courtship behaviour before laying the spermatophores. A male newt fans his tail, creating current that wafts his cloacal pheromones towards the female’s nose and then lays up to eight spermatophores on the substrate [63]. Salamanders’ sexual behaviour is well documented. To persuade the female to take up one or more spermatophores the pair engages in a tail-straddle walk [64]. At this point the male delivers Acps to the female.

The delivery of Acps in salamanders can occur by (1) ‘pulling’ and ‘snapping’, (2) ‘snapping’, (3) ‘slapping’ and (4) ‘biting’. (1) When pulling the male rapidly scrapes his chin a few times on the female’s body while pressing down; this occurs for example in *Desmognathus ocoee*[65]. When he scrapes the female’s back, his premaxillary tooth wounds her skin superficially, and at the same time, his mental gland swabs the wounded area. The substance from the male gland most likely diffuses into the female’s circulatory system via the capillaries that are abundant in the skin of this lungless salamander [66]. (2) ‘Snapping’ consists of one very forceful stroke, which the male administers by a sudden snapping action of his body [67]. It delivers male gland secretions via the same principle as the ‘pulling’ or scraping behaviour. (3) The third delivery mode is called ‘slapping’, and occurs in species with relatively large mental glands, that lack protruding maxillary teeth. The males of these species slap their mental gland on the female’s snout, so the female inhales the secretion through her nose [66,68-70]. The slapping of the mental gland on the female’s nose results in delivery of pheromones to the vomeronasal organ (a well-developed chemosensory structure inside the nose of salamanders) and thus to the accessory olfactory system [70]. (4) The fourth chemical delivery mode, the ‘biting’, has been observed only in *Desmognathus wrighti*[71] and *Desmognathus aeneus*[64]. The male bites the female with his highly modified mandibular teeth, and powerfully holds on. The secretions from his mental gland reach the female’s blood through perforations in her skin caused by his bite. The mental glands of *D. wrighti* and *D. aeneus* are unique among salamanders in that they open into the male’s mouth, instead of externally on his chin [64].

Irrespective of these modes of delivery of these salamander Acps, the observed female response is the same: the secretion significantly increases female receptivity during courtship and reduces insemination time [72,73]. In all tested species, pairs in which the female had received mental gland secretion engaged in courtship interactions more quickly than pairs in which the female had not received such substances because the mental gland were previously ablated [66]. Subsequently, it was shown that one of the mental gland’s active Acps is a cytokine-like protein, called the Plethodonthid Receptivity Factor (PRF) [72], which increases female receptivity [74]. In addition, Plethodontid Modulating Factor (PMF) and Sodefrin Precursor-like Factor (SPF) have been shown to be involved in reduced time to insemination [73]. Hence, these Acps play an important role in male mating success.

## Conclusions

### Importance of the evolution of alternative delivery strategies of Acps for sexual selection

The reviewed examples of unconventional Acps transfer via piercing, stabbing and stinging have several things in common. Firstly, they all result in the injection of Acps, usually directly into the hemolymph or blood. Secondly, the actions of the Acps indicate that they favour the mating success of the sperm donor. Thirdly, in all the reviewed cases, no Acps have been found to be transferred along with the sperm, though clear evidence of the lack of Acps in the ejaculates has not been provided. However, the mode of sperm transfer seems not to allow for Acps transfer via the conventional route, i.e., the ejaculate. This implies that due to their mode of sperm transfer, these animals all transfer Acps by injecting them directly through the partner’s body wall, rather than by adding them to their sperm. Similar methods of injection of Acps are not found in animals with conventional sperm transfer. Hence, this synthesis seems broadly applicable to the widely diverse taxa reviewed here (Table 1).

**Table 1:** Summary of the taxa mentioned in this review

**Ejaculate transfer**	**Taxa**	**Injecting device**	**Accessory gland**	**Effect**	**Reference**
Skin groove	Oligochaeta	Copulatory setae	Setal gland	Sperm uptake	[33,34]
Skin groove	Opistobranchia	Penial stylet	Prostate gland	Unilateral mating?	[36,44]
Encased	Pulmonata	Love dart	Digitiform gland	Sperm storage, remating inhibition	[44,49]
Encased	Arachnida	Stinging organ	Venomous gland	Subdue aggressive behaviour?	[58]
Encased	Amphibia	Premaxillary teeth	Mental gland	Spermatophore uptake	[58,65]

The modes via which the spermatozoa are transferred, the devices injecting the Acps, the glands producing the Acps, and their effects in the receiver are included. Question marks indicate that the effect has not been fully confirmed.

The transfer of Acps via injection is independent of whether the ejaculate is transported externally over a groove on the body wall (e.g., earthworms, sea slugs), or packaged (e.g., terrestrial snails, scorpions, salamanders). The used injection devices (e.g., love dart, copulatory setae, premaxillary teeth) are clearly structures that are specialised for the transfer of the substances that are produced in their accompanying accessory glands.

Acps are an important aspect of mating systems, especially when individuals mate multiply and/or store sperm. Despite the clearly independent evolution of the injection devices, our review illustrates that these odd delivery modes all cause behavioural and/or physiological change in their recipients that benefit the reproductive success of the sperm donor. We tentatively suggest that the pattern observed in the reported cases is mainly driven by post-copulatory selection, as it may increase the chance and/or efficacy of fertilization when facing rival sperm.

The list of animals with unconventional modes of Acps transfer is growing, but currently limited to several relatively well-studied taxa. Importantly, such substances constitute a reproductive strategy in both separate sexes and hermaphroditic animals; hence given that the injection of Acps has been found in such a wide range of taxonomic groups, we expect these modes of Acps delivery to be more widespread than currently realised.

The described findings now equip us with predictions about in which types of sperm transfer one can expect to find similar peculiar behaviours. For instance, animals with dissociated sperm transfer do not seem to represent the best candidate for the identification of alternative delivery of Acps. In such a mating system males deposit their sperm in the environment for females to pick up without meeting the males [26]. Thus, there is no contact between the sexes during sperm transfer, which prevents males of such species from coercing females into using their sperm. In such situations, chemical cues associated with the spermatophore are most likely involved at the stage prior to sperm uptake [26,27]. Moreover, females of these species have been shown to take up only one spermatophore and not store sperm [75,76], implying that post-copulatory sperm competition is absent.

However, indirect sperm transfer and/or copulation may exist in different degrees within an order or genus (e.g., collembolans and water mites: [28]). Hence, we do by no means intend to exclude particular animal groups from further identification of alternative delivery of Acps. Rather, we encourage researchers to collect more data on male reproductive behaviour in groups displaying indirect (e.g., collembolans, mites, millipedes, bryozoans) or non-conventional sperm transfer (e.g., leeches, bedbugs, strepsipterans), keeping in mind that post-copulatory process are the ultimate target of Acps.

To conclude, empirical progress in the assessment of agents transferred separate from the sperm, offers the possibility to gain better insight into the evolution of male reproductive strategies. Hence, studying the functional significance of alternative Acps transfer, may lead to a deeper understanding of the selective pressure to increase male fertilization success.

## Abbreviations

Acps: Accessory gland products; PMF: Plethodontid Modulating Factor; PRF: Plethodonthid Receptivity Factor; SPF: Sodefrin Precursor-like Factor.

## Competing interests

The authors declare that there are no competing interests.

## Authors’ contributions

ZVZ and JMK conceived and wrote the manuscript. IS contributed to the literature survey and to drafting the manuscript. All authors read and approved the final manuscript.
